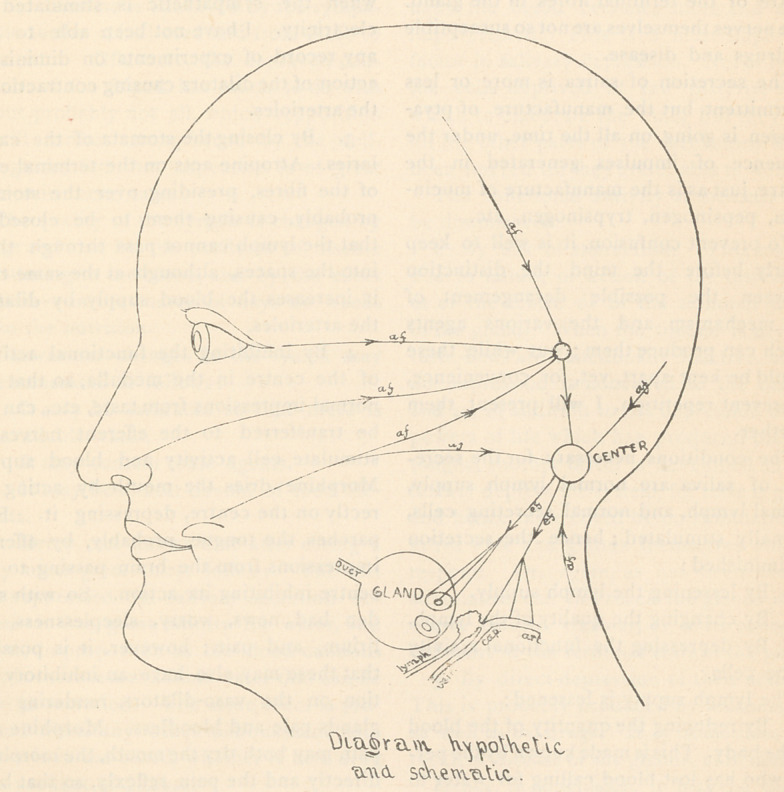# Pathology and Treatment of a Dry Tongue

**Published:** 1888-11

**Authors:** W. C. Caldwell

**Affiliations:** 168 South Halsted Street


### PATHOLOGY AND TREATMENT OF A DRY TONGUE.

BY W. C. CALDWELL, M. D.

Probably a more scientific title for this paper would be "The pathology and Treatment of Diminished Secretion from the Glands Connected with the Buccal Cavity," but the one given seems the more expressive, because everyone recognizes that a dry tongue is a symptom of profound depression of the powers of life. "The Pathology and Treatment of the Typhoid State" would be equally as expressive, but it is too broad and includes more than I desire to write about at this time.

I will present the subject in the following order:

I. Causation;
II. Pathological Significance;
III. Pathology; and,
IV. Treatment.

### I. CAUSATION.

The three possible ways in which the tongue may become dry are either by increased evaporation from its surface, by diminished movement of the tongue, or by diminished secretion of saliva. The first two, no doubt, are factors in its production whenever the mouth is open and whenever the tongue lies motionless for hours unbathed in buccal secretions, as occurs in coma and in the typhoid state, the patient lying with his mouth open and his tongue motionless.

In health, if the tongue be held motionless, breathing through the mouth, there will be perceptible dryness in even ten minutes, so these can be factors in its production.

Coma does not usually continue longer than twenty-four hours without a dry tongue. Dickinson (Lumleian Lectures, 1888), does not think this due simply to the air passing over it, and cites as proof that where an otherwise healthy individual has the nostrils plugged there is no such dryness. This, though, does not prove it, because unlike in coma, the individual keeps the tongue moist by occasionally moving it about in the mouth; however, in coma I think it probable that not only is the tongue dry because it is motionless, but very likely there is some action on the centre in the medulla, which will be considered later.

But, undoubtedly by far the most important factor in the production of a dry tongue is diminished secretion from those glands which pour their elaboration into the buccal cavity, namely, the salivary, buccal, palatine, lingual, malar, and labial glands.

To determine how much diminished secretion has to do with causing the tongue to become dry, Dickinson has made many experiments on the parotid gland by catheterization. He says that "A tube passed into one of these channels in health could be made to drip saliva abundantly by the application of acetic acid to the tongue. When the tongue was dry it was usually impossible to obtain any by such means. With advanced and complete dryness I have never been able to get a drop; with lesser dryness often a little."

### II. PATHOLOGICAL SIGNIFICANCE.

I have thought it well to say a few words about the grave pathological significance of a dry tongue as a means of impressing the great importance of its treatment. The gravity of this condition is made manifest by the fact that every one regards a dry tongue as a very unfavorable sign, and does all in his power to forestall its approach.

It is an unfavorable sign:

1. Because it retards digestion, and

2. Because it is evidence of profound derangement of the powers of life.

Diminished salivary secretion interferes with digestion in a number of ways, and perhaps the easiest way to show this is to give its functions, and then the effect of its absence will be more easily made apparent.

1. The saliva, by moistening the dry food, enables the tongue to roll it into a bolus which is easily swallowed; hence, when the patient has a dry mouth solid food is swallowed with great difficulty and is contra-indicated for this as well as other reasons.

2. On account of the law of chemistry that acids and alkalies are mutually attracted, it follows that when the bolus entering the stomach is saturated with alkaline saliva, the gastric juice rapidly penetrates it and promotes its digestion.

3. The ptyalin of the saliva converts starch into sugar, and in proportion as the saliva is diminished is this act interfered with. But probably it is not so much the loss of this conversion of starch into sugar as it is the effect it has upon the taste of the food, which in turn, as will be shown, stimulates further secretion.

4. But in addition to this means of stimulating taste, one of the most important functions of the saliva is to keep the taste-buds moist so that they can perform their specific function, namely, that of making eating pleasant and thus inducing us to eat, and also that of exciting to functional activity the salivary and gastric glands for the secretion of the digestive fluids.

Physiologically the secretion of saliva is stimulated reflexly by thoughts of food, by sight and smell, but mainly by taste when the food is introduced into the mouth. In sleep the senses convey no impressions to the centre; hence, during sleep very little saliva is secreted. Also, when the tongue is dry, when the epithelial covering and taste-buds are parched and shriveled, there is no sense of taste; and hence, equally so, there are no impressions conveyed to the centre to stimulate the secretion of saliva. The patient with a dry tongue has no taste, no appetite, and when food is placed in the mouth there is no response from the salivary glands.

5. But equally as important as the stimulation of the buccal glands is the synchronous stimulation of the gastric glands. While the luscious morsel is being rolled in the mouth, the gastric secretion preparatory for its further digestion in the stomach is stimulated reflexly by sense of taste; hence, when the tongue is dry, not only are the salivary glands at rest, but the stomach, receiving no influence ahead, is idle, unprepared, and surprised by an untimely visitor. Instead of meeting a rosy mucous membrane, with clear drops of gastric juice trickling down its walls, the dry, neutral, unchanged bolus comes in contact, possibly, with a pale, bloodless surface.

To the person in health, whose body is strong and vigorous, and who has a large amount of reserve vitality, such an interference with digestion for a short time might not make a great deal of difference, but to the patient struggling with some wasting or long-continued disease, whose powers of life are already taxed to their utmost—whose heart possibly is beating at the rate of 150 per minute, unable to stand the smallest fraction of further strain—to such a patient every ounce of blood pabulum absorbed is of the greatest importance to his life.

Hence, any interference with digestion when the powers of life are running low—when, on account of the adynamic condition, threatened dissolution is impending—is of the gravest moment; and it is usually when dissolution overhangs the patient, when he most needs good digestion, that the tongue is dry as a board seasoned in an August sun.

Too much cannot be said of the great importance of keeping the tongue moist in those self-limited acute diseases which have become asthenic, such as typhoid fever, pneumonia, scarlet fever, etc. A few ounces more of nourishment absorbed may determine the fate of the patient in whom we can almost hear the death-rattle. By this nourishment, during a few hours, he may be tided over the impending collapse and begin convalescence.

A dry tongue, also, is evidence of profound derangement of the powers of life, because, not only is the salivary secretion suppressed, but the same cause very likely suppresses the gastric, pancreatic, and biliary secretions; also it is evidence of grave changes in the blood, the great food supply for all the tissues.

III. PATHOLOGY.

The morbid anatomy of a dry tongue will not be noticed, but instead, the morbid physiology, which produces it by diminishing the buccal secretions. These secretions are produced by a complicated mechanism, consisting of numerous parts, any one of which becoming impaired, the secretion may be diminished or entirely suppressed. The pathology is nothing more than a description of the morbid actions of this mechanism and their cause, by which the secretion is either diminished, or increased, or changed in quality. For obvious reasons, in this paper only the pathology of diminished secretion will be considered; this being the main cause of a dry tongue, and often having a common origin with those other grave symptoms, indicating the typhoid state. A thorough knowledge of its mechanism will naturally form the data from which to deduce the rational indications for the treatment of that part of the typhoid state. But in order to understand properly morbid action of this mechanism, it will be necessary to study first this mechanism and its means of normal excitation; then we will be prepared to understand the ways in which it can be deranged and the agents which may impair it.

In presenting this, the following order will be used:

1. The mechanism of salivary secretion and its physiological excitation;

2. Possible derangement of the mechanism, which can diminish the secretion; and,

3. The various agents, including drugs and the morbid materials of disease, which, acting on different parts of the mechanism, may diminish the secretion of saliva.

For the production of any thing, there must be the raw material and the mechanism for its elaboration. In the production of saliva the essential elements may be classed as follows:

1. Secreting cells.

2. Lymph.

3. Nervous mechanism presiding over the intracellular metabolism and lymph supply.

This complicated secreting mechanism consists of secreting cells, adjacent to which are lymph spaces, containing the raw material which has been poured into them through stomata from the capillaries, and over the action of the cells and the lymph supply preside two nerve mechanisms—a central and a peripheral. The sources of this nerve supply are from both the sympathetic and cranial nerves, but for simplicity no description of the two sources will be given; neither will the peripheral mechanism be described. For all practical purposes in this paper this will not be necessary. The accompanying diagram, used to illustrate this mechanism, is entirely schematic, no attempt being made to give the anatomy. In it the peripheral mechanism is not shown, neither is there a separate representation of the sympathetic. The nervous mechanism, shown in the diagram, consists of afferent and efferent nerves and a centre in the medulla.

The afferent nerves convey impressions to the centre from taste, smell, sight, thoughts, and also from irritation of sensory nerves in various other parts of the body. The efferent nerves, probably six kinds in all, convey impulses from the centre to the glands. The trophic fibres continually convey impulses generated in the centre to regulate cellular growth and nutrition.

Probably ptyalinogenic (anabolic) fibres continuously convey impulses generated in the centre to regulate that continuous protoplasmic metabolism, the manufacture of ptyalinogen, for the intermittent production of ptyalin. The secreting fibres at intervals convey reflex impulses from the centre, producing secretory activity of the cells. The vaso-dilator and vaso-constrictor fibres preside over the arterioles, regulating their calibre and the quantity of blood passing through them. Probably regulating fibres preside over the stomata in the capillaries determining the quantity of lymph which can pass through them into the lymph spaces.

The centre in the medulla receives impressions from the afferent nerves and switches them onto the efferent nerves, by which influence the size of the arterioles and stomata are regulated, and also the activity of secretion; in addition, the centre generates impulses which pass along the trophic and ptyalinogenic fibres to the gland.

Physiologically the mechanism does not act except when it is set to work by reflex influence from taste, smell, sight and thoughts of savory food. Normally, when there is no reflex stimulation, there is no secretion. The normal excitation of the mechanism is illustrated by the accompanying diagram, in which the afferent nerves are shown conveying impressions from taste, etc., either directly or indirectly, to the centre in the medulla, from which they are reflected to the efferent nerves, along which they travel to the gland. This

Diagram hypothetic and schematic.

is the explanation of why the hungry boy's mouth waters when he sees the inviting pastry and cake in the baker's window, or when his nostrils are regaled by the exhilarating aroma arising from the steaming dinner-pot. These physiological facts can be used to advantage in retaining and creating an appetite in the invalid. When there is any interference in any part of the circuit from the afferent ends to the terminal fibres in the glands, the secretion is proportionally deranged. Hence, if from any cause, the circuit is broken in any part the normal reflex stimulation of saliva can not occur. And if this be continued long the mouth and tongue become dry The points at which disease and drugs usually break the circuit are the taste-buds, the centre or the terminal fibres in the gland. The nerves themselves are not so susceptible to drugs and disease.

The secretion of saliva is more or less intermittent, but the manufacture of ptyalinogen is going on all the time, under the influence of impulses generated in the centre, just as is the manufacture of mucinogen, pepsinogen, trypsinogen, etc.

To prevent confusion, it is well to keep clearly before the mind the distinction between the possible derangement of the mechanism and the various agents which can produce them; but while these should be kept apart, yet, for convenience, to prevent repetition, I will present them together.

The conditions necessary for the secretion of saliva are normal lymph supply, normal lymph, and normal secreting cells, normally stimulated; hence the secretion is diminished:

1. By lessening the lymph supply.

2. By changing the quality of the lymph.

3. By depressing the functional activity of the cells.

The lymph supply is lessened:

1. By reducing the quantity of the blood in the body. This is made known by the person who has lost blood calling for water to quench his thirst The tongue becomes dry in profuse diarrhœa and where there is free perspiration.

2. By contracting the arterioles so that a smaller quantity of blood passes through the gland, and hence less lymph enters the spaces, the food reservoir of the secreting cells. The calibre of the arterioles can be reduced either by increased action of the constrictors, or diminished action of the dilators.

While physostigma stimulates the secreting cells, it also stimulates the constrictors; hence, while at first it increases the secretion, later, as soon as the stock of lymph in the spaces is used up, the flow of saliva is diminished because the arterioles are contracted. The same phenomenon occurs when the sympathetic is stimulated by electricity. I have not been able to find any record of experiments on diminished action of the dilators causing contraction of the arterioles.

3. By closing the stomata of the capillaries. Atropine acts on the terminal ends of the fibres, presiding over the stomata probably, causing them to be closed so that the lymph cannot pass through them into the spaces, although at the same time it increases the blood supply by dilating the arterioles.

4. By inhibiting the functional activity of the centre in the medulla, so that the normal impressions from taste, etc., can not be transferred to the efferent nerves to stimulate cell activity and blood supply. Morphine dries the mouth by acting directly on the centre, depressing it. Fear parches the tongue, probably, by afferent impressions from the brain passing to the centre inhibiting its action. So with sudden bad news, worry, sleeplessness, delirium, and pain; however, it is possible that these may also have an inhibitory action on the vaso-dilators, rendering the glands pale and bloodless. Morphine and pain may both dry the mouth, the morphine directly and the pain reflexly, so that both render the centre functionally inactive, yet a small dose of morphine, by relieving the pain, and, hence, the reflex inhibition, may moisten the mouth.

It is probable in apoplexy that the pressure affects the centre and helps to cause the dry tongue. Though sometimes disease of the brain increases the secretion. Stimulation of the cortex cerebri of a dog near the sulcus cruciatus sometimes is followed by secretion (Landois). Pulling out a loop of intestine inhibits the reflex secretion of saliva (Pawlow). The afferent impression is conveyed to the medulla, where it inhibits the action of the centre. It is very likely that partly in this way is the dry tongue in typhoid fever produced. The irritation from the ulcerated and tympanitic intestine is conveyed by the sympathetic to the spinal cord and from there to the centre. Of course, the depraved condition of the blood has much to do with it, but probably not all, which supposition is still further strengthened by the well-recognized fact that turpentine is of great service in these cases.

The quality of the lymph is changed by drugs and also by the products of disease circulating in the blood, and by the effect they have in the course of days or weeks upon the nutrition.

The various changes in the lymph may be enumerated as follows:

1. It may be saturated so that there is no available water.

2. It may contain toxic agents.

3. It may be almost devoid of food elements.

1. Every one knows that after eating a salty meal great thirst follows. The salt has suppressed the salivary secretion because it has great affinity for water, and hence, in proportion as the blood contains salt, is there less available water that can be used in secretion. The same effect is produced when any other deliquescent substance is in the blood, whether it be a drug or a product of disease.

It is very probable that the excessive quantity of histolytic waste circulating in the blood of the fever patient has much to do with his thirst, and, later, his dry tongue, though, of course, the evaporation on account of the high temperature has its effect in producing thirst, just as we become thirsty on a hot day after freely perspiring.

2. Just as some of the vegetable alkaloids and mineral drugs introduced into the circulation act as antisialogogues, so it is probable that some of the numerous leucomaines produced by the various diseases act as antisialogogues.

3. After the patient has been sick for some time, the digestive organs, along with the others having suffered, they now in turn increase the trouble further by their inability to supply sufficient food. Gradually the blood becomes more and more impoverished. This is a most important factor in salivary depression. Not only are the cells deprived of raw material from which to elaborate the fluid, but they and all the other parts of the mechanism have been starved till they are unable to work.

The functional activity of the cells may be diminished:

1. By lack of stimulation. This may be due to failure of action of the taste-buds or of the centre or of the terminal fibres in the gland. When the tongue is dry the taste-buds are functionally inactive, hence no impression is conveyed to the centre, and so, in addition to the depression of the powers of life which has produced the dry tongue, another factor is developed to still further depress the system, for taste is gone, and hence there could be no stimulation of saliva were the glands themselves able to respond. Or, again, as has been shown, stimulation can not reach the glands if the centre fails to transfer, or if its course is cut short at the terminal ends in the gland.

3. By direct depression of their activity. This is probably produced by leucomaines as well as by drugs. It is sometimes the result of disease of the glands, as in mumps.

Hence, in conclusion, it may be postulated that in order that there may be physiological secretion there must be:

1. Normal secreting cells.

2. Normal nervous mechanism—end organs, centre and terminal fibres intact, so that there is no break in the circuit.

3. Proper supply of blood, containing sufficient amount of nourishment and water and free from antisialogogue leucomaines.

And it may also be postulated with equal positiveness, when the tongue is dry from diminished secretion, that there is derangement of some one of them. Either the secreting cells are deranged, or the blood supply is imperfect or poisoned, or the nervous mechanism is not intact.

### IV. TREATMENT.

The object of treatment is to improve digestion so that the vital organs may be fed, for without nourishment they can not work long in health, and much less when they are already wasted, weakened, and grappling with some malignant disease.

The rational indications for treatment are learned by a survey of the possible points of the mechanism that can be deranged, and then a determination of what point is at fault. If the centre is at fault, and treatment be applied empirically at the periphery, little success can be expected from such a course.

The thing to do is to find the defective point and repair it, if possible, just as the watchmaker looks for the broken piece and replaces it.

The indications for treatment based on the pathology may be classified as follows:

1. To improve the lymph supply.
2. To improve the quality of the lymph.
3. To improve the functional activity of the secreting cells.

As the different parts of the organism are so closely related and dependent on each other, the improvement of one often improves another; hence, in filling one indication sometimes another may be satisfied, so there is no sharp line of demarkation between these indications.

### QUANTITY OF LYMPH.

1. If the dry tongue follows profuse hæmorrhage, of course replenish with water and liquid food.

If lymph is deficient from excessive discharge, as occurs in diarrhœa where the stools are copious and watery, control the diarrhœa or soon another factor of disease will appear—failure of digestion from suppressed secretions. If it be already present, in addition to checking the alvine discharge, attention should be directed to keeping the buccal cavity moist, to avoid further adynamia and collapse. For this, see treatment of the taste-buds. If the quantity of water in the blood is deficient on account of excessive evaporation during high temperature, allow the thirsty patient to drink water freely to refill the empty vessels, unless from some cause it should produce vomiting.

2. If, from any source, impressions travel to the centre, which either inhibit its action, or, when switched on to the efferent fibres in some of the possible ways, inhibit secretion, then give such drugs as either prevent such impressions being conveyed to the centre, or depress the centre so that they will not be transferred to the glands.

If the patient with parched tongue is sleepless and tosses to and fro on his uncomfortable bed, or worse, if he is delirious, give bromides, hydrate of chloral, urethan, or some other hypnotic, to arrest the pathological cerebration which is conveyed to the centre and interferes with secretion. This is the explanation of why the dry tongue of the delirious patient, after a few hours' refreshing sleep, sometimes begins to moisten.

For the same reason, if, in a continued and low grade of disease, there is much pain, morphine judiciously given will often moisten the dry tongue. Also, for the same reason, partly, turpentine is of such great benefit where there is inflammation of the mucous membrane of the intestine, with dry tongue.

The value of turpentine in chronic intestinal catarrh, with dry, glazed tongue, also in typhoid fever, is well recognized by everyone. Turpentine is an antiseptic and arrests fermentation, removing the tympanites; it also relieves intestinal congestion by giving tonicity to the intestinal circulation, by improving the cutaneous capillary circulation, and by lessening the catarrhal inflammation or the ulcerated condition of

Peyer's glands, the impressions are shut off which, transmitted to the centre, inhibit secretion. But in addition to this means of moistening the tongue, turpentine undoubtedly does much as a diffusible stimulant, acting on the heart and vaso-motor centre, raising the blood-pressure and producing a sense of exhilaration. Yet, without its action on the intestinal canal, it would fall far short of its present position in the treatment of typhoid fever.

If the function of the centre is compromised by pressure from cerebral hæmorrhage or inflammatory exudate, of course all that can be done is to treat the disease.

QUALITY OF THE LYMPH.

1. If there is deficient quantity of available water in the blood on account of saturation with hygroscopic, pathological, retrograde metamorphoses, give water freely, not only to supply the need, but to wash out the vascular and lymph channels, clearing away as fast as possible the débris from the glands.

2. At present not much is known of the physiological action of leucomaines, but if the secretion is suppressed on account of the blood being charged with antisialogogue leucomaines, inhibiting the centre, stimulating the constrictors, paralyzing the dilators, closing the stomata, or depressing the secreting cells, in lieu of any thing better till further research has shed more light, exhibit plenty of water to wash them out of the system through the kidneys, skin, and intestines.

Also,bathe the fever patient one or more times daily, to promote the action of the sweat-glands. For other reasons, add one-third alcohol to the water for the bath.

Water taken internally is of unquestionable service in disease. When large quantities are taken, large amounts pass out of the organism through the excretory glands, and with it is carried the retrograde products soluble in water; also those rendered soluble by the action of medicine. Water also washes out the intestinal canal, removing irritant material, and preventing reabsorption of the waste excreted through the intestinal glands. When given with medicines which are to act after entering the blood, it prevents irritation and promotes their absorption. When taken after the food it promotes absorption of the peptones, and hence increases further digestion. The peptones, like all products of fermentation, arrest the succession of complicated reactions which take place in the process. Water increases cellular metabolism, especially in the weak, whose cells have a low grade of vitality, and in those depraved by disease. Water is necessary for the metabolism of all cells, but in those cells of poor vitality, water is a tonic. It greatly increases their functional activity, increasing both the production and excretion of urea, sulphuric and phosphoric acids. In these weak cells it probably increases their nutrition by washing out the waste and supplying needed water.

But too much water is said to do harm. It may cause dyspepsia by unduly diluting the gastric juice, or, if the stomach is irritable, it may produce vomiting. It is also said to destroy the red corpuscles when the blood is diluted too much.

Yet it seems to me that there is far more danger of using too little than too much. Undoubtedly, when there is a high and continued fever, rapidly melting down the nitrogenous structures and filling the blood with urea and urates, that the free, frequent, and continuous exhibition of water is indicated to prevent asthenia, and if it is already present, to aid in removing it.

3. If the blood is so profoundly depraved that it is destitute of proper materials to supply the cells with sufficient food, in addition to treating the disease, every effort should be made to improve the digestion. This will be noticed a little later. This may accomplish very little, but that little may be sufficient to sustain the heart and respiratory function a few hours longer.

FUNCTIONAL ACTIVITY OF THE CELLS.

Very common causes of depressed functional activity of the secreting cells are lack of stimulation on account of the dry, functionally inactive taste-buds, and lack of food from failure of digestion in general.

Much can be done to improve the taste-buds by acting locally. Keep the gums, teeth, and tongue well cleansed by frequent washing with some appropriate antiseptic wash, which puts the epithelial covering of these structures in as healthy a condition as possible. Instead of giving plain water, allow the patient to have acidulated drinks, because the acid stimulates secretion from the alkaline mucous membrane.

Where the tongue is dry, brown, encrusted, and hard, remove the coat as much as possible, and for a few minutes before giving liquid nourishment, place some crushed ice on it, and allow it to remain. The ice will at first cause anæmia, which will soon be followed by a local hyperæmia, resulting in increased nutrition of the taste-buds. The tongue for the time being will be red and moist. If this be persisted in, in typhoid fever, pneumonia, and other low fevers, however little effect in itself it may have, it will often improve digestion enough, so that in a few days the improved digestion is sufficient to keep the tongue moist, and thus in turn again improve digestion and the powers of life.

But probably, of any single agent for improving digestion when the tongue is dry, the most potent is some acceptable, pleasant-tasting preparation of alcohol.

In the typhoid state this should never be given alone, but always with liquid food, the explanation of which will soon follow. Alcohol is superior to the other diffusible stimulants for continuous exhibition in many ways. Of course turpentine has its unique place when the intestinal canal is a cause of the dry tongue. But no other drug can take the place of alcohol in the treatment of the typhoid state. It is pleasant to take, and does not interfere with digestion by repugnance to it. It produces hyperæmia of the mucous membrane, and both directly and reflexly increases the digestive secretions. At the same time reflexly, and a little later directly, it dilates the cerebral arterioles, causing a feeling of exhilaration, which is also increased by simultaneous stimulation of the heart, so that the tired, exhausted patient, under the excitement, has more couarge to take nourishment.

If alcohol were given alone, without food, it would stimulate the heart and produce hyperæmia of the digestive canal. This expenditure of energy would be wasted, for there would be no food in the canal to be digested. Hence, in giving alcohol to sustain the heart, always give food. Alcohol and liquid food should be given together in small quantities every two hours.

Alcohol occupies the peculiar position of being a stimulant, as ether, chloroform, and turpentine, and at the same time of being a food, as such carbo-hydrates as starch and sugar. Like ether, it liberates the energy which during health has been stored up, and, like the carbo-hydrates, it supplies force itself by being oxidized, and in addition to this, while it is itself oxidizable, it has the remarkable property of diminishing oxidation of the tissues. It arrests oxidation of the tissues by rendering the hæmoglobin less active, and by abstracting the oxygen from the tissues—usually stopping the oxidation process at the stage of fat.

Alcohol is a valuable agent in the treatment of the typhoid state, because it improves digestion, sustains the heart, supplies force, lessens oxidation, and quiets the restless brain.

Not only when the tongue is dry, but before the system has been so much depressed, in continued disease, where the patient is on liquid diet for days, the food should be seasoned with proper quantity of salt. This is specially overlooked when the patient is on milk diet. Of course, there is some salt in milk, just as there is in meat, but not enough, except in normal mother's milk, where the babe only needs the salt it contains. However, when cow's milk is used, either for the babe or for the sick, it should be salted, the salt preventing solid coagulation of the cow's milk by the gastric juice. Salt has important uses in digestion and nutrition. It is necessary for the secretion of the hydrochloric acid, which in fevers is usually suppressed long before the peptic secretion. It is also used in the production of the bile salts, and that portion which enters the blood, in some way is necessary to the vital processes in producing urea and carbonic acid.

When the tongue persistently remains dry, in spite of all these means, the frequent administration in small quantity of alcohol and peptonized beef, milk, or gruel, which requires no digestive help from the crippled salivary and gastric glands, may be slowly absorbed by the sluggish circulation in the gastric mucous membrane in limited quantity, but sufficient to furnish a little nourishment to the starving secreting cells, which may be manifested by a slight moistening of the tongue and exfoliation of the dead coat.

It is not only important to remove, but equally so to prevent the development of, a dry tongue, and in attempting to do this no pains should be spared in using all those apparently and singly insignificant means of exciting taste and increased desire for food. Relishes and aromatics should be judiciously used in flavoring the food to suit the peculiar whims of the patient. It should be remembered that smell and sight, and the thoughts which they suggest, are often powerful agents in their action on the digestive tract, advantage of which should be taken, preparing the meals so as to be as inviting and as little repugnant as possible. Here is a chance for the trained nurse to show her taste, judgment, and culinary ability. She who, by her tidiness, taste, and good cooking, caters successfully to the capricious appetite of the fever-stricken patient, does as much for the sufferer as the physician.

The conclusion to be reached from the above is, that in continued fevers, while medicines can be of unquestionable value, yet, without the copious use of water and palatable, nutritious liquid food, at short and regular intervals, they are of little value.

168 South Halsted Street.

## Figures and Tables

**Figure f1:**